# Effects of Feeding Fermented *Medicago sativa* (Plus Soybean and DDGS) on Growth Performance, Blood Profiles, Gut Health, and Carcass Characteristics of Lande (Meat) Geese

**DOI:** 10.3389/fphys.2022.902802

**Published:** 2022-07-13

**Authors:** Hui Li, Yang Liu, Lan Wei, Qian Lin, Zhifei Zhang

**Affiliations:** ^1^ College of Agronomy, Hunan Agricultural University, Changsha, China; ^2^ Institute of Bast Fiber Crops, Chinese Academy of Agricultural Sciences, Changsha, China; ^3^ College of Animal Sciences and Technology, Hunan Agricultural University, Changsha, China; ^4^ Hunan Institute of Animal Science and Veterinary Medicine, Changsha, China

**Keywords:** alfalfa, mixed silage, serum biochemical indices, goose, antioxidative capacity, intestinal development

## Abstract

The objective was to investigate the effects of alfalfa (*Medicago sativa Linn*)-mixed silage fermentation material (AMSFM) on various aspects of growth, function, and carcass characteristics of Lande (meat) geese. Based on a previous study, we used the following AMSFM: 80% Alfalfa +10% soybean meal +10% DDGS ensiled for 45 days. Lande geese, n = 264, 77 days of age, were randomly allocated into four groups with six replicates in each group. Control geese were fed a basal diet, whereas experimental groups were fed a basal diet supplemented with 6, 12, or 24% AMSFM. The experiment lasted 21 days. The AMSFM promoted some aspects of growth, with increase (*p* < 0.05) in leg muscle rate, lean meat rate, muscle protein content, and total energy content of leg muscle plus concurrent decreases (*p* < 0.05) in crude fat content and abdominal fat rate in chest muscle. In addition, AMSFM increased (*p* < 0.05) glutathione content in chest and leg muscles and serum superoxide dismutase activity, and it reduced (*p* < 0.05) muscle malondialdehyde content and serum concentrations of triglycerides, total cholesterol, urea, and aspartate aminotransferase, consistent with good liver and kidney function. Moreover, AMSFM improved (*p* < 0.05) ileum morphology. In conclusion, the optimal supplemented rate of AMSFM in the meat geese diet (12%) improved immunity and antioxidant status and enhanced growth performance and carcass characteristics of meat geese.

## 1 Introduction

Poultry production is increasing, but traditional protein sources, including soybean and fish meal, are becoming less available and more expensive, prompting the need for alternatives. Alfalfa (*Medicago sativa L.*) is termed the “king of forage” due to its high yield and that it contains a favorable nutrient content and various active substances (Chen et al., 2020; Ni et al., 2020). Ensiling alfalfa simplifies its preservation and reduces feed costs (Chen et al., 2013) and minimizes competition with humans for food sources (Wang and Yu, 2020). In addition, alfalfa-mixed silage is easy to produce and is a very good feed source (Plaizier, 2004; Wang et al., 2019).

Meat geese are efficient herbivores, capable of consuming more crude fiber and less grain and generating high economic value for meat, liver, and feathers (Gao et al., 2016). However, there are few reports on feeding alfalfa-mixed silage to meat geese. The objective was to investigate the effects of alfalfa (*Medicago sativa Linn*)-mixed silage fermentation on various aspects of growth, function, and carcass characteristics of Lande (meat) geese.

## 2 Materials and Methods

### 2.1 Experimental Materials

Based on previous studies, the alfalfa-mixed silage fermentation material (AMSFM) used was 80% alfalfa +10% soybean meal +10% DDGS. Alfalfa was collected from the alfalfa planting base of Hunan Deren Animal Husbandry Technology Co., Ltd. It was cut on sunny days at the budding stage, processed to a straw length of <1 cm, mixed with other ingredients, and seal-silaged for 45 days. The nutritional analysis of AMSFM is in Table 1.

**TABLE 1:** Nutrient composition of alfalfa-mixed silage fermentation material (AMSFM).

Item	Content in AMSFM
DM (% FW)	35.41
CP (%DM)	28.44
NDF (%DM)	28.01
ADF (%DM)	16.84
CF (%DM)	17.71
ASH (%DM)	6.06
EE (%DM)	5.28
GE (MJ/kg)	17.17
Ca (%)	0.61
P (%)	0.17

DM, dry matter; CP, crude protein; NDF, neutral detergent fiber; ADF, acid detergent fiber; CF, crude fiber; Ash, Ash content; EE, ether extract; GE, gross energy; Ca, calcium; P, phosphorus.

### 2.2 Experimental Design

This study was conducted at the Institute of Bast Fiber Crops, Chinese Academy of Agricultural Sciences. Lande geese (n = 264), 77 days old, were randomly allocated into four groups. Each group had six replicates (11 geese per replicate). Control geese (CONT) were fed a basal diet, whereas experimental groups were fed a basal diet supplemented with 6, 12, or 24% AMSFM (TRT6, TRT12, and TRT24, respectively), with *ad lib* access to feed and water. The animal test was conducted at the Shiji Lake Animal Test Base (112.38 ^0^E, 28.82 ^0^N) in Yuanjiang, Institute of Hemp, Chinese Academy of Agricultural Sciences. Durations of the preexperimental and trial periods were 7 and 21 days, respectively, with geese maintained under conditions with good ventilation and natural light. Immunization and disinfection were done according to standard procedures. Diets (Table 2) were prepared with reference to National Research Council (1994) requirements for meat geese.

**TABLE 2:** Composition and nutrient levels of basal diets for meat geese (air-dry basis, %). Control geese (CONT) were fed a basal diet, whereas TRT6, TRT12, and TRT24 were fed a basal diet supplemented with 6, 12, or 24% alfalfa-mixed silage fermented material, respectively.

Item
	CONT	TRT6	TRT12	TRT24
Ingredient	—			
Corn	45.70	45.95	44.84	40.00
Soybean meal	18.34	20.17	21.57	24.05
Defatted rice bran	15.00	10.00	6.90	0.80
Wheat bran	15.00	10.00	6.90	0.80
Rice husk powder	0.00	0.90	1.45	2.45
AFSM	0.00	6.00	12.00	24.00
Soybean oil	2.70	2.60	2.91	4.35
limestone	1.00	0.90	0.90	0.90
CaHPO_4_·2H_2_O	0.95	1.13	1.13	1.22
98.5% methionine	0.00	0.00	0.00	0.03
78.5% lysine	0.01	0.00	0.00	0.00
NaCl	0.30	0.30	0.30	0.30
1% premix^1)^	1.00	1.00	1.00	1.00
Total	100	100	100	100
Nutrient levels^2)^	—			
ME (Mcal/kg)	2.70	2.70	2.70	2.70
CP(%DM)	15.62	15.63	15.62	15.62
CF(%DM)	4.69	4.70	4.68	4.69
Ca (%)	0.66	0.66	0.65	0.66
Available P (%)	0.36	0.37	0.36	0.36
Lys	0.81	0.81	0.82	0.83
Met	0.26	0.26	0.26	0.27
Met + Cys	0.55	0.54	0.53	0.54

The premix provided the following (per kilogram of complete diet) microelements: VA, 12000 IU; VD1 2500 IU; VE, 20 mg; VK3 3 mg; VB1 3 mg; VB2 8 mg; VB5 7 mg; VB12 0.03 mg; d-pantothenic acid 20 mg; nicotinic acid 50 mg; biotin 0.1 mg; folic acid 1.5 mg; Cu (as copper sulfate) 9 mg; Zn (as zinc sulfate) 110 mg; Fe (as ferrous sulfate) 100 mg; Mn (as managanese sulfate) 100 mg; Se (as sodium selenite) 0.16 mg; and I (as potassium iodide) 0.6 mg.

Nutrient levels are calculated values.

AFSM, alfalfa-mixed silage fermentation material.

### 2.3 Sample Collection

At the end of the experiment (105 days of age), the total weight of geese in each replicate was determined and recorded. On the 21st day of the experiment, geese were fasted for 6 h, and one goose was randomly selected from each replicate (a total of 6 geese in each treatment group) and weighed. Blood (5 ml) was collected by venipuncture of a wing vein, using a 10-ml syringe, put into centrifuge tubes, and after 30 min, centrifuged at ? x g for 10 min. Thereafter, serum was separated and stored at −20°C. Then, selected geese were killed by cutting the carotid artery and dissected. Chest and leg muscles were excised to determine meat nutrient quality and antioxidant indexes. The duodenum, jejunum, and ileum were identified and excised and a 1.5-cm portion of the middle of each intestinal segment was recovered, washed with 0.9% normal saline, and placed in a 50 ml test tubes containing formaldehyde.

### 2.4 Measurements and Methods

#### 2.4.1 Growth Performance

The body weight of experimental geese was determined on Days 1 and 21 of the formal experiment. Throughout the experimental period, delivered and residual feed were weighed and recorded daily. Growth, feed intake, and feed efficiency were determined as follows:

$$\text{Average daily feed intake}\left(\text{g}\right)=\;\frac{\text{total feed intake}}{\text{ test days}},$$

$$\text{Average daily gain}\left(\text{g}\right)=\;\frac{\text{final weight}-\text{initial weight}}{\text{ test days}},$$

$$\text{Feed weight ratio}=\;\frac{\text{average daily feed intake}}{\text{average daily gain}}.$$

#### 2.4.2 Carcass Characteristics

Another goose was selected from each replicate to determine post-slaughter status. The goose was weighed, slaughtered, and feathers removed. Then, we determined the following weights: slaughter, semi-clean bore, full clean bore, abdominal fat, the chest muscle, the leg muscle, the spleen, the bursa, the thymus, the liver, the muscular stomach, the glandular stomach, and viscera. We used the following formulas:

$$\text{Slaughter rate}\left(\text{\%}\right)=\;\frac{\text{weight after slaughter }}{\text{weight before slaughter}}\times 100\mathrm{\%,}$$

$$\text{Half clean bore rate }\left(\text{\%}\right)=\;\frac{\text{half clean bore weight}}{\text{weight before slaughter}}\times 100\mathrm{\%,}$$

$$\text{Full clean bore rate }\left(\text{\%}\right)=\;\frac{\text{weight of both pectoral muscles}}{\text{total net bore weight ∗ }1000}\times 100\mathrm{\%,}$$

$$\text{Pectoral muscle rate }\left(\text{\%}\right)=\;\frac{\text{full clean bore weight}}{\text{weight before slaughter}}\times 100\mathrm{\%,}$$

$$\text{Leg muscle rate }\left(\text{\%}\right)=\;\frac{\text{weight of both leg muscles}}{\text{full clean bore weight }\times 1000}\times 100\mathrm{\%,}$$

$$\text{Lean meat rate }\left(\text{\%}\right)=\;\frac{\text{chest muscle weight }+\text{leg muscle weight}}{\text{full clean bore weight }\times 1000}\times 100\mathrm{\%,}$$

$$\text{Organ index}\left(\text{g}/\text{kg}\right)=\;\frac{\text{ fresh weight of internal organs}}{\text{weight before slaughter}}\times 100\mathrm{\%,}$$

$$\text{Abdominal fat rate }\left(\text{\%}\right)=\;\frac{\text{abdominal fat }+\text{ muscle and gastric peripheral fat}}{\text{total net bore weight }\times 1000}\times 100\%.$$

#### 2.4.3 Muscle Characteristics

Portions (∼15 g) of pectoral and leg muscles were excised from each sample goose, processed to a constant weight with a freeze-drying machine, and dry matter contents measured. Crude protein (CP) was determined by the Kjeldahl method, crude fat was extracted by the Soxhlet method, and crude ash was determined by a high-temperature (550°C) burning method. Total energy contents were determined by a 5E calorimeter.

#### 2.4.4 Antioxidant Indexes

Portions (0.15–0.2 g) of chest and leg muscle tissue samples were excised and placed into 2 ml homogenization tubes, with 9 times the volume of precooled normal saline and sterilized homogenization beads added. The samples were then centrifuged at 3000 x g for 15 min at 4°C and tissue supernatants collected. Total antioxidant capacities (T-AOC), malondialdehyde (MDA) and glutathione (GSH) contents, catalase (CAT) and superoxide dismutase (SOD) activities of chest and leg muscles as well as serum T-AOC, MDA content, GSH content, CAT activity, SOD activity, and glutathione peroxidase (GSH-Px) activity were measured.

#### 2.4.5 Serum Indexes

The Mairui bs-420 Automatic Biochemical Instrument was used to determine serum physiological and biochemical indexes, including serum concentrations of glucose (Glu), triglyceride (TG), total cholesterol (CHO), urea (urea), uric acid (UA), albumin (ALB), total protein (TP), globulin (GLB) and creatinine as well as the activities of alanine aminotransferase (ALT) and aspartate aminotransferase (AST).

#### 2.4.6 Intestinal Morphology

After fixation, intestinal tissue samples were dehydrated through an alcohol series, embedded in paraffin, sectioned (5 μm), stained with H&E, and observed under an optical microscope. Five intact villi and crypts were selected from each slice in independent visual fields, and the thickness of each segment, from the top of the villi to the opening of the crypt was measured and recorded as villi height (VH). Furthermore, the distance from the recess opening to the recess base was recorded as the recess depth (CD). The ratio of VH to CD was recorded as V/C.

### 2.5 Data Analyses

Data were analyzed with ANOVA, with LSD used to locate differences. All analyses were done with SPS 19.00, *p* < 0.05 was considered significant, and results were reported as mean ± SD.

## 3 Results

### 3.1 Growth Performance

There were no differences (*p* > 0.05) among groups for final weight, ADG, ADFI, or F/G (Table 3).

**TABLE 3:** Effects of alfalfa-mixed silage fermentation material (AMSFM) on growth performance of meat geese. Control geese (CONT) were fed a basal diet, whereas TRT6, TRT12, and TRT24 were fed a basal diet supplemented with 6, 12, or 24% AMSFM, respectively.

Team	CONT	TRT6	TRT6	TRT24	*p value*
Initial weight (kg)	3.81 ± 0.02	3.82 ± 0.01	3.82 ± 0.01	3.81 ± 0.02	0.313
Final weight (kg)	4.06 ± 0.13	4.21 ± 0.11	4.09 ± 0.32	4.08 ± 0.21	0.717
ADG (g/d)	14.43 ± 2.90	18.38 ± 4.14	17.30 ± 5.16	17.52 ± 5.27	0.624
ADFI (g/d)	182.29 ± 14.40	182.26 ± 20.51	178.86 ± 9.93	174.63 ± 21.01	0.845
F/G	13.7 ± 3.25	11.39 ± 2.58	10.93 ± 2.27	10.42 ± 1.64	0.352

Values in the table are mean ± SD (n = 11).

ADG, average daily gain; ADFI, average daily feed intake; F/G, feed weight ratio, ADFI/ADG.

### 3.2 Carcass Characteristics

The leg muscle ratio was lower (*p* = 0.010) in CONT compared to the other three groups (Table 4). The abdominal fat ratio in TRT24 was lower *p* = 0.012) than that in TRT6 or CONT and the lean meat ratio in TRT24 exceeded (*p* < 0.05) that of the CONT.

**TABLE 4:** Effects of alfalfa-mixed silage fermentation material (AMSFM) on carcass characteristics of meat geese. Control geese (CONT) were fed a basal diet, whereas TRT6, TRT12, and TRT24 were fed a basal diet supplemented with 6, 12, or 24% AMSFM, respectively.

	CONT	TRT6	TRT12	TRT24	*p value*
Carcass ratio (%)	88.72 ± 3.02	89.79 ± 4.65	89.22 ± 11.01	89.22 ± 1.58	0.993
Semi-eviscerated ratio (%)	79.69 ± 7.33	82.53 ± 4.68	81.15 ± 0.88	80.48 ± 3.39	0.758
Eviscerated ratio (%)	67.41 ± 2.85	70.90 ± 4.39	67.55 ± 3.10	67.33 ± 3.32	0.269
Chest muscle ratio (%)	13.12 ± 1.40	13.89 ± 1.68	13.93 ± 1.02	14.91 ± 2.10	0.374
Leg muscle ratio (%)	10.61 ± 1.03^b^	12.26 ± 0.84^a^	12.31 ± 1.44^a^	13.19 ± 0.64^a^	0.010
Abdominal fat ratio (%)	3.83 ± 0.62^a^	3.75 ± 0.58^a^	3.30 ± 0.58^ab^	2.65 ± 0.50^b^	0.012
Lean meat ratio (%)	23.73 ± 2.12^b^	26.10 ± 1.23^ab^	26.19 ± 1.52^ab^	28.31 ± 2.51^a^	0.018

Values in the table are mean ± SD (n = 11).

^a,b^Pairs of means with the same superscript letter within the same row are not significantly different at least at the p > 0.05 level.

### 3.3 Muscle Characteristics

The content of DM in chest muscle was lower (*p* = 0.049) in TRT24 than in CONT (Table 5). The content of CP in chest muscle was higher (*p* = 0.039) in TRT12 than in CONT. The contents of EE in the chest muscle of CONT were higher (*p* = 0.003) than in the other three groups. In leg muscle, DM content was higher (*p* = 0.041) in TRT12 than in CONT or TRT24, CP content was greater (*p* = 0.012) in TRT12 or TRT24 than in CONT, and GE content was higher (*p* = 0.023) in TRT12 compared to CONT or TRT24.

**TABLE 5:** Effects of alfalfa-mixed silage fermentation material (AMSFM) on muscle characteristics of meat geese. Control geese (CONT) were fed a basal diet, whereas TRT6, TRT12, and TRT24 were fed a basal diet supplemented with 6, 12, or 24% AMSFM, respectively.

Item		CONT	TRT6	TRT12	TRT24	*p value*
**Chest muscle**	**DM (%FW)**	25.06 ± 0.98^a^	24.28 ± 1.04^ab^	24.48 ± 0.55^ab^	23.67 ± 0.42^b^	0.049
	**CP (%DM)**	80.64 ± 2.80^b^	85.64 ± 1.51^ab^	92.33 ± 8.72^a^	86.85 ± 4.81^ab^	0.039
	**EE (%DM)**	13.22 ± 1.83^a^	10.29 ± 1.74^b^	10.54 ± 1.60^b^	8.68 ± 1.11^b^	0.003
	**ASH (%DM)**	1.63 ± 0.48	2.04 ± 0.24	1.92 ± 0.08	2.07 ± 0.15	0.693
	**GE (MJ/kg)**	21.70 ± 0.79	21.81 ± 0.70	21.96 ± 0.32	21.96 ± 0.76	0.889
**Leg muscle**	**DM (%FW)**	24.11 ± 1.57^b^	25.97 ± 2.29^ab^	27.14 ± 1.96^a^	24.70 ± 1.29^b^	0.041
	**CP (%DM)**	74.26 ± 2.23^b^	76.98 ± 2.39^ab^	79.83 ± 3.47^a^	80.76 ± 3.41^a^	0.012
	**EE (%DM)**	18.19 ± 6.12	17.83 ± 4.62	17.77 ± 3.56	15.88 ± 5.98	0.890
	**ASH (%DM)**	1.16 ± 0.64	1.39 ± 0.47	1.13 ± 0.23	0.72 ± 0.10	0.465
	**GE (MJ/kg)**	23.26 ± 0.51^b^	24.20 ± 1.01^ab^	25.37 ± 1.13^a^	23.99 ± 1.22^b^	0.023

Values in the table are mean ± SD (n = 11).

^a,b^Pairs of means with the same superscript letter within the same row are not significantly different at least at the p >0.05 level.

DM, dry matter; CP, crude protein; EE, ether extract; Ash, Ash content; GE, gross energy.

### 3.4 Serum Indexes

Serum concentrations of triglycerides and urea were highest in CONT (*p* = 0.011 and *p* = 0.003, respectively), whereas serum AST activity was highest (*p* = 0.005) in CONT (Table 6).

**TABLE 6:** Effects of alfalfa-mixed silage fermentation material (AMSFM) on serum biochemistry of meat geese. Control geese (CONT) were fed a basal diet, whereas TRT6, TRT12, and TRT24 were fed a basal diet supplemented with 6, 12, or 24% AMSFM, respectively.

Item	CONT	TRT6	TRT12	TRT24	*p value*
**GLU (mmol/L)**	9.09 ± 1.10	9.21 ± 1.06	8.95 ± 1.36	9.72 ± 0.72	0.766
**TG (mmol/L)**	1.33 ± 0.32^a^	0.87 ± 0.26^b^	0.78 ± 0.02^b^	0.72 ± 0.13^b^	0.011
**CHO (mmol/L)**	5.79 ± 1.28	5.09 ± 0.44	5.12 ± 0.58	5.52 ± 1.38	0.720
**UREA (mmol/L)**	2.11 ± 0.15^a^	1.62 ± 0.20^b^	1.57 ± 0.15^b^	1.64 ± 0.20^b^	0.003
**UA (μmol/L)**	0.15 ± 0.03	0.15 ± 0.03	0.12 ± 0.04	0.14 ± 0.04	0.683
**ALB (g/L)**	12.53 ± 1.44	13.14 ± 1.69	12.58 ± 1.87	12.88 ± 1.10	0.939
**TP (g/L)**	50.10 ± 3.12	54.27 ± 9.29	46.35 ± 7.79	50.12 ± 7.95	0.537
**GLB (g/L)**	37.57 ± 1.89	41.13 ± 7.78	33.77 ± 6.70	37.24 ± 7.09	0.466
**ALT (U/L)**	10.28 ± 1.56	9.93 ± 1.21	9.68 ± 0.71	10.80 ± 2.38	0.771
**AST (U/L)**	38.27 ± 1.94^a^	31.78 ± 3.76^b^	30.33 ± 1.81^b^	29.40 ± 1.14^b^	0.005
**Creatinine (μmol/L)**	5.08 ± 0.98	4.76 ± 0.72	4.78 ± 0.78	5.35 ± 0.62	0.751

Values in the table are mean ± SD (n = 11).

^a,b^Pairs of means with the same superscript letter within the same row are not significantly different at least at the p >0.05 level.

GLU, glucose; TG, triglyceride; CHO, total cholesterol; UREA, UREA; UA, uric acid; ALB, albumin; TP, total protein; GLB, globulin; ALT, Glutamic-pyruvic Transaminase; AST, glutamic oxalacetic transaminase; Creatinine, Creatinine.

### 3.5 Muscle Antioxidant Status

In the chest muscle, the MDA concentration was higher (*p* = 0.014) in CONT versus TRT6 or TRT12, GSH was lowest (*p* < 0.010) in CONT, and CAT was highest (*p* = 0.023) in TRT6 and TRT12 (Table 7). In the leg muscle, MDA was lowest (*p* = 0.040) in TRT6 and TRT12 and GSH was higher (*p* = 0.022) in TRT12 and TRT24 than in CONT.

**TABLE 7:** Effects of alfalfa-mixed silage fermentation material (AMSFM) on antioxidant indexes of chest and leg muscles from meat geese. Control geese (CONT) were fed a basal diet, whereas TRT6, TRT12, and TRT24 were fed a basal diet supplemented with 6, 12, or 24% AMSFM, respectively.

Team	CONT	TRT6	TRT12	TRT24	*p value*
			Chest muscle		
**MDA (nmol/mL)**	0.72 ± 0.14^a^	0.50 ± 0.14^bc^	0.44 ± 0.12^c^	0.66 ± 0.13^ab^	0.014
**T-AOC (U/mL)**	0.09 ± 0.02	0.10 ± 0.02	0.10 ± 0.03	0.09 ± 0.01	0.775
**GSH (umol/L)**	1.91 ± 0.44^b^	2.96 ± 0.33^a^	3.01 ± 0.44^a^	3.35 ± 0.94^a^	<0.010
**CAT (U/mL)**	0.80 ± 0.17^b^	1.21 ± 0.26^a^	1.15 ± 0.17^a^	1.01 ± 0.22^ab^	0.023
**SOD (U/mL)**	21.54 ± 5.06	25.50 ± 7.77	22.87 ± 4.52	22.54 ± 5.87	0.695
	**Leg muscle**				
**MDA (nmol/mL)**	0.39 ± 0.08^a^	0.26 ± 0.06^b^	0.22 ± 0.08^b^	0.29 ± 0.07^ab^	0.040
**T-AOC (U/mL)**	0.16 ± 0.04	0.17 ± 0.04	0.19 ± 0.05	0.19 ± 0.05	0.609
**GSH (umol/L)**	2.46 ± 1.04^c^	3.25 ± 0.57^bc^	3.99 ± 1.24^ab^	4.69 ± 0.96^a^	0.022
**CAT (U/mL)**	0.97 ± 0.19	1.06 ± 0.18	0.98 ± 0.13	1.07 ± 0.26	0.738
**SOD (U/mL)**	23.07 ± 5.13	25.63 ± 5.19	26.23 ± 6.13	24.41 ± 4.89	0.810

Values in the table are mean ± SD (n = 11).

^a–c^Pairs of means with the same superscript letter within the same row are not significantly different at least at the p >0.05 level.

T-AOC, total antioxidant capacity; MDA, malondialdehyde; GPX, glutathione content; CAT, catalase; SOD, superoxide dismutase.

### 3.6 Serum Antioxidant Status

Regarding serum antioxidant capacity, the SOD activity was greater (*p* = 0.047) in TRT6 and TRT12 than in CONT (Table 8).

**TABLE 8:** Effects of alfalfa-mixed silage fermentation material (AMSFM) on serum antioxidant indexes in meat geese. Control geese (CONT) were fed a basal diet, whereas TRT6, TRT12, and TRT24 were fed a basal diet supplemented with 6, 12, or 24% AMSFM, respectively.

Team	CONT	TRT6	TRT12	TRT24	*p value*
**MDA (nmol/mL)**	9.57 ± 2.23	7.86 ± 1.14	6.65 ± 2.30	9.12 ± 2.69	0.272
**T-AOC (U/mL)**	0.99 ± 0.33	1.21 ± 0.32	1.03 ± 0.26	1.06 ± 0.33	0.667
**GSH (umol/L)**	9.12 ± 3.61	10.01 ± 1.46	10.71 ± 3.57	9.21 ± 2.11	0.759
**CAT (U/mL)**	0.07 ± 0.02	0.09 ± 0.02	0.07 ± 0.01	0.07 ± 0.02	0.217
**SOD (U/mL)**	630.53 ± 120.87^b^	847.04 ± 191.04^a^	804.78 ± 87.20^a^	756.34 ± 69.28^ab^	0.047
**GSH-P**_**X**_ **(nmol/mg prot)**	934.53 ± 202.41	1045.36 ± 129.37	1211.87 ± 244.26	1143.44 ± 184.81	0.168

Values in the table are mean ± SD (n = 11).

^a,b^Pairs of means with the same superscript letter within the same row are not significantly different at least at the p >0.05 level.

T-AOC, total antioxidant capacity; MDA, malondialdehyde; GPX, glutathione content; GSH-Px, glutathione peroxidase; CAT, catalase; SOD, superoxide dismutase.

### 3.7 Small Intestine Histomorphological Indexes

**FIGURE 1:**
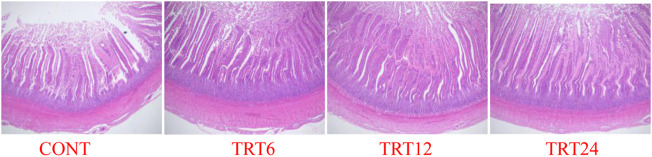
Light micrograph of jejunum morphology in meat geese (40 × multiple). Control geese (CONT) were fed a basal diet, whereas TRT6, TRT12, and TRT24 were fed a basal diet supplemented with 6, 12, or 24% alfalfa-mixed silage fermented material, respectively.

**FIGURE 2:**
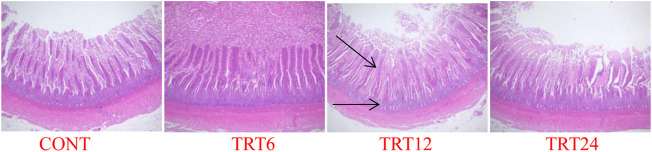
Light micrograph of ileum morphology in meat geese (40 × multiple). Control geese (CONT) were fed a basal diet, whereas TRT6, TRT12, and TRT24 were fed a basal diet supplemented with 6, 12, or 24% alfalfa-mixed silage fermented material, respectively.

**FIGURE 3:**
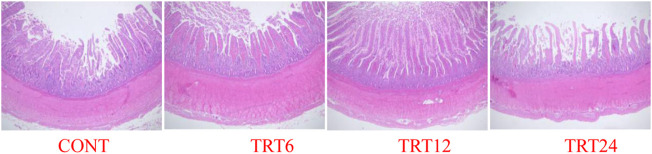
Light micrograph of duodenum morphology in meat geese (40 × multiple). Control geese (CONT) were fed a basal diet, whereas TRT6, TRT12, and TRT24 were fed a basal diet supplemented with 6, 12, or 24% alfalfa-mixed silage fermented material, respectively.

Effects of AMFSM on small intestine histology of meat geese are shown in Table 9 and Figures 1–3. In the ileum, TRT12 had the least (*p* = 0.022) CD and the highest (*p* = 0.038) V/C ratio.

**TABLE 9:** Effects of alfalfa-mixed silage fermentation material (AMSFM) on intestinal histomorphological indexes in meat geese. Control geese (CONT) were fed a basal diet, whereas TRT6, TRT12, and TRT24 were fed a basal diet supplemented with 6, 12, or 24% AMSFM, respectively.

Location		CONT	TRT6	TRT12	TRT24	*p value*
**Jejunum**	**VH (μm)**	1083.79 ± 172.04	1331.91 ± 143.99	1266.09 ± 202.10	1110.42 ± 211.26	0.293
	**CD (μm)**	227.07 ± 9.05	253.67 ± 33.86	245.18 ± 21.90	236.13 ± 21.87	0.441
	**V/C**	4.78 ± 0.77	4.68 ± 1.23	4.60 ± 1.02	4.69 ± 0.67	0.994
**Ileum**	**VH (μm)**	977.96 ± 45.47^b^	1197.35 ± 107.65^a^	1210.25 ± 37.82^a^	1088.33 ± 103.28^ab^	0.012
	**CD (μm)**	237.19 ± 7.35^a^	245.14 ± 17.81^a^	216.22 ± 9.90^b^	229.38 ± 2.98^ab^	0.022
	**V/C**	4.57 ± 0.56^b^	4.76 ± 0.62^b^	5.60 ± 0.11^a^	4.54 ± 0.56^b^	0.038
**Duodenum**	**VH (μm)**	954.21 ± 190.83	1040.42 ± 101.76	1081.33 ± 278.37	1005.42 ± 89.56	0.850
	**CD (μm)**	248.13 ± 35.31	197.99 ± 28.94	220.13 ± 14.81	240.38 ± 39.12	0.152
	**V/C**	4.18 ± 0.45	4.80 ± 0.85	4.40 ± 1.13	4.22 ± 0.44	0.702

Values in the table are mean ± SD (n = 11).

^a,b^Pairs of means with the same superscript letter within the same row are not significantly different at least at the p >0.05 level.

VH, villus height; CD, crypt depth; V/C, villus height/crypt depth.

## 4 Discussion

Compared to the CONT, geese consuming AMSFM had ∼2% more rapid ADG and ∼2% higher final weight, although there were no significant differences among groups for either endpoint. Similarly, in a previous study (Xu et al., 2021), there were no significant differences in the ADG and ADFI of meat geese when adding biofermented feed at the later growth stage. Perhaps weight development of meat geese decreases at a later growth stage, and the basal diet met the needs of growth and development so that the fermented feed had no significant impact on growth performance. In previous studies, adding alfalfa to the diet promoted animal growth (Hu et al., 2021), with fermented alfalfa better than alfalfa meal (Yin and Zhou, 2015). Fermentation can improve nutritional quality and palatability, and enhance digestion, absorption, and growth performance. Adding a fermented feed to a goose diet enhanced the composition of goose intestinal flora, which improved nutritional status and intestinal health, and enhanced the growth performance of meat geese (Yan et al., 2019). Furthermore, feeding grass powder had no significant effect on the slaughter performance of geese, but reduced fat deposition, (Jiang et al., 2012), similar to current results.

In this experiment, the leg muscle ratio, abdominal fat ratio, and lean meat ratio of the three AMSFM groups were significantly lower than those of the control group. Perhaps AMSFM improved feed nutritional value and utilization. AMSFM is rich in amino acids and effective components such as alfalfa polysaccharide, saponins, and carotene, which could improve the metabolic rate of protein, crude fat, and other substances, promote digestion and absorption of nutrients, reduce fat deposition, and improve lean meat yield (Francisco et al., 2020).

The CONT geese had the lowest CP content in both chest and leg muscles; this was attributed to alfalfa, DDGS, and soybean meal in AMSFM being rich sources of protein and amino acids. Furthermore, the range of water content of chest and leg muscles was 72.86%–76.33%, similar to a previous report (71.32%–77.20%) from five breeds of geese (Tang et al., 2010). The water content of muscle is directly related to the taste, juiciness, and chewiness of meat, with implications for the economic value of meat (Biesek et al., 2020). The EE of the chest muscle was highest in the CONT, consistent with a reduction in fat content in geese supplemented with AMSFM. Perhaps the alfalfa saponins, fatty acids, and other substances in AMSFM reduced the crude fat content of chest muscle in meat geese.

Serum TG concentrations were significantly higher in CONT geese than those in the other three groups. In a previous study, alfalfa meal significantly reduced TG in animals (Li et al., 2019). Furthermore, lower serum urea concentrations in geese supplemented with AMSFM were consistent with greater incorporation of nitrogen into muscle. In addition, significantly lower serum AST concentration in geese supplemented with AMSFM indicated good liver function, consistent with previous studies (Li et al., 2015; Yang et al., 2022).

After fermentation, portions of saponins, flavonoids, vitamins, and other probiotics in AMSFM were converted into amino acids that could be effectively absorbed by livestock and poultry, maintaining the dynamic balance of amino acids in the animal body, and improving antioxidant capacity (Gungor et al., 2021; Lu et al., 2021). Organic acids produced during fermentation removed oxygen free radicals and enhanced antioxidative ability (Zhan et al., 2017). Antioxidant peptides are formed by fermentation of bioactive substances, which could prevent peroxidation, and protect the normal structures and functions of various tissues and organs as well (Choi et al., 2010). CAT, SOD, and GSH-Px decompose hydrogen peroxide and remove free radicals, serving as the main endogenous antioxidative enzymes to protect the body from oxidative damage (Fang et al., 2002).

After slaughter, numerous reactive oxygen species are produced, affecting meat color and lipid peroxidation in the muscle. As antioxidant substances in the muscle are limited, meat quality eventually decreases (Echegaray et al., 2021). In previous studies, feeding alfalfa saponins tended to reduce MDA concentrations in layers and increased activities of GSH-Px and SOD (Fan et al., 2018). In a comparison of adding alfalfa meal or fermented alfalfa to the diet of geese, fermented alfalfa had better effects in improving antioxidant performance, attributed to the greater content of vitamins, alfalfa polysaccharides, isoflavones, and small molecule peptides (Pleger et al., 2020). Overall, these effects were similar to those in the present study.

The small intestine is not only critical for poultry to digest and absorb nutrients but also the largest immune organ in the body. It constitutes the first barrier to preventing invasion of pathogenic bacteria. Intestinal health is one of the most important indicators to reflect poultry health (Chen et al., 2013). The functions of villi and crypt in the small intestine are to promote digestion and absorption of nutrients. Intestinal crypt cells differentiate into villi to replace shed or damaged villous cells (Woyengo et al., 2010). The higher the villus height and the ratio of the villus to the crypt in the morphology of the small intestine, the larger the surface area and the greater the nutrient absorption capacity (Jazi et al., 2017). Missotten et al. (2013) reported that feeding broilers fermented feed improved the villus height and the ratio of the villus to the crypt, and decreased crypt depth in their small intestine. Li et al. (2020) added fermented soybean meal to the diet of broilers and improved the duodenum and jejunum. In this study, AMSFM significantly improved ileal morphology, consistent with the previous studies. With the increase of the AMSFM proportion, the villus height and the villus ratio of the small intestine increased first and then decreased, the crypt depth gradually decreased, and the thickness of the intestinal wall also decreased, which helped the transportation and absorption effect of nutrients. Therefore, AMSFM had positive effects on the intestinal structure of meat geese, and promoted nutrient absorption and utilization, with 6–12% AMSFM having the best effects. In addition, there were some microbial metabolites, e.g., butyric acid after fermentation of alfalfa polysaccharide (Flint et al., 2008), and organic acids, amino acids, and small peptides produced by the metabolism of other substances, acting on the intestine and inducing the proliferation and differentiation of intestinal cells, which also improved the biological environment and maintained the balance and health of intestinal flora (Zahran et al., 2014; Jazi et al., 2018).

## 5 Conclusions

Adding AMSFM to the diet of meat geese improved some aspects of growth performance, lean meat ratio, and muscle protein, and reduced the fat content in muscle. Moreover, dietary AMSFM improved the antioxidant capacity and immune organ index, improved the morphological structure of the ileum, and promoted good liver and kidney function. In this study, supplementing the diet of meat geese with 12% AMSFM provided the best overall response.

## Data Availability

The raw data supporting the conclusions of this article will be made available by the authors, without undue reservation.
